# Stress‐to‐Light Conversion in an Earth‐Abundant Oxide Semiconductor

**DOI:** 10.1002/advs.75587

**Published:** 2026-05-08

**Authors:** Tomoki Uchiyama, Koki Otonari, Reona Omori, Guangfa Yang, Eiji Nishibori, Ying Chen, Xu‐Guang Zheng, Chao‐Nan Xu

**Affiliations:** ^1^ Department of Material Science and Engineering Faculty of Engineering Tohoku University Sendai Miyagi Japan; ^2^ Department of Material Science and Engineering Graduate School of Engineering Tohoku University Sendai Miyagi Japan; ^3^ Department of Physics Faculty of Pure and Applied Sciences Tsukuba Research Center for Energy Materials Science Hydrogen Boride Research Center (HBRC) Tsukuba Institute for Advanced Research (TIAR) University of Tsukuba Ibaraki Japan; ^4^ Global Learning Center Tohoku University Sendai Miyagi Japan; ^5^ Department of Physics Faculty of Science and Engineering Saga University Saga Japan

**Keywords:** rare‐earth‐free, self‐powered photonics, stress‐to‐light conversion, zinc oxide

## Abstract

Stress‐to‐light conversion in solids represents a unique photonic functionality, yet it has never been realized in a chemically simple and sustainable material. Zinc oxide (ZnO) is an earth‐abundant compound widely used in cosmetics, food supplements, paints, and medicinal products since prehistoric times. It is also a promising semiconductor for electronics and photonics owing to its high transparency, high electron mobility, and wide bandgap, more than three times that of silicon. Here, we show that the sustainable semiconductor ZnO exhibits strong near‐infrared (NIR) luminescence under elastic stress when defect‐engineered to stabilize a *p*‐type state. This transformation overcomes the intrinsic *n*‐type character of ZnO through the partial substitution of Zn^2+^ with Li^+^ or Na^+^, introducing deep‐level defects that enable stress‐driven NIR emission and ferroelectricity. These coupled electronic and structural effects reveal a previously unknown light‐emitting function in a simple oxide lattice. Our findings establish a rare‐earth‐free, self‐powered platform for NIR photonics, offering scalable opportunities for biophotonic signaling and infrastructure health monitoring.

## Graphical abstract

Stress‐to‐light conversion in solids represents a unique photonic functionality, yet it has never been realized in a chemically simple and sustainable material. Here, we show that sustainable semiconductor ZnO exhibits strong near‐infrared (NIR) luminescence under elastic stress when defect‐engineered to stabilize the *p*‐type state. Our findings establish a rare‐earth‐free, self‐powered platform for NIR photonics, offering scalable opportunities for biophotonics.

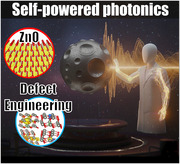

## 1 Introduction

Stress‐to‐light conversion—the direct transduction of mechanical stress into light—offers a unique route for light sources, wireless sensing, and photonic functionalities. The phenomenon underlying this concept, known as mechanoluminescence (ML), has been widely studied, yet most reported cases involve fracture or friction (fractoluminescence and triboluminescence) [1, 2], which require material damage and limit durability. In contrast, elastic ML, driven by reversible lattice strain, represents a sustainable form of stress‐to‐light conversion without structural failure. This effect was first demonstrated in SrAl_2_O_4_:Eu^2+^ and ZnS:Mn^2+^ [3, 4, 5], representing a unique class of photonic responses arising from the coupling among mechanical deformation, charge carrier dynamics, and excited electronic states. Since then, ML has been widely explored for wireless lighting, stress sensing, infrastructure monitoring, and biophotonics [6, 7, 8, 9, 10, 11, 12, 13, 14, 15, 16, 17, 18, 19, 20]. However, strong elastic ML typically requires complex compositions and rare‐earth dopants, limiting scalability and industrial use [21, 22, 23, 24, 25, 26, 27, 28, 29]. Zinc oxide (ZnO), an earth‐abundant semiconductor with a wide bandgap, has long been considered promising for electronics and photonics. Yet, light emission triggered by mechanical stress has never been realized in this simple oxide. Here, we report stress‐driven near‐infrared (NIR) elastic ML in defect‐engineered *p*‐type ZnO, challenging the long‐standing view that pure ZnO cannot exhibit ML. Partial substitution of Zn^2+^ with Li^+^ or Na^+^ introduces deep‐level defects that stabilize *p*‐type conduction and couple elastic strain to photon emission. This design enables strong, self‐powered emission within the first biological window (650–900 nm) under gentle mechanical stimulation, including ultrasound. This rare‐earth‐free system opens new pathways toward implantable health monitors, biophotonic communication tools, and smart infrastructure sensors with ultrahigh sensitivity to microstrains.

## 2 Results and Discussion

### 2.1 Microstructural Characterization

**FIGURE 1:**
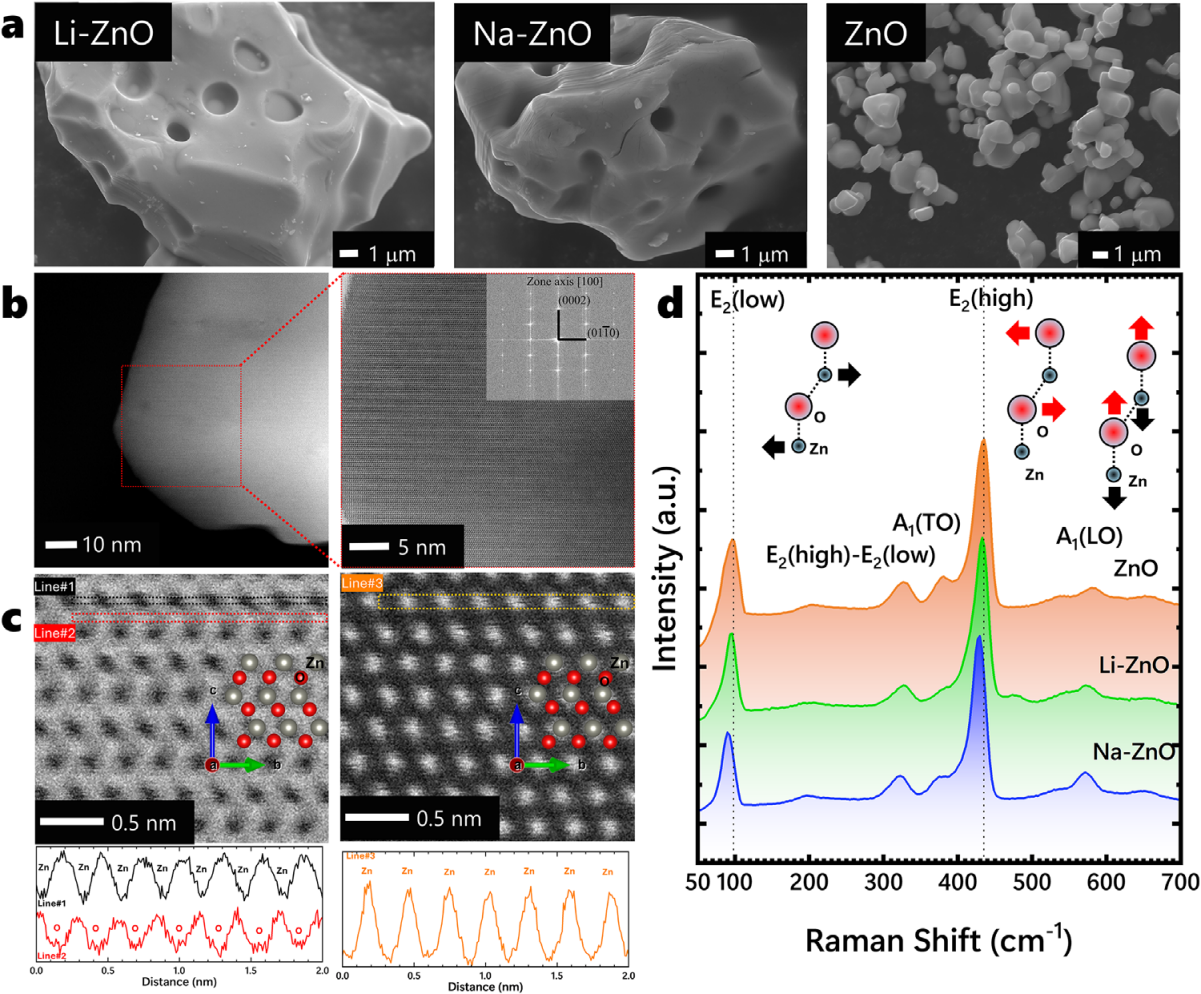
Structure and morphology of defect‐engineered ZnO. (a) SEM images showing enhanced crystal growth and characteristic crater‐like surface features in 1.6 mol.% Li‐ and 3 mol.% Na‐doped ZnO compared with undoped ZnO. (b) Low‐magnification STEM‐HAADF image of Li‐ZnO with the corresponding FFT pattern (inset), indexed to the (0002) and (10‐10) reflections of wurtzite ZnO. (c) High‐resolution STEM‐ABF (left) and STEM‐HAADF (right) images along the *bc* crystallographic plane. Line‐profile analysis across atomic columns identifies Zn (profiles #1 and #3) and O (#2). (d) Raman spectra of Na‐ZnO (blue), Li‐ZnO (green), and undoped ZnO (orange), showing systematic *E*_2_ mode shifts and enhanced *A*_1_(LO) intensity, indicative of defect formation.

X‐ray diffraction (XRD) analysis of ZnO samples prepared as described in the Experimental Section revealed that Li^+^ and Na^+^ ions were incorporated into Zn sites in the wurtzite structure (denoted as Li‐ZnO and Na‐ZnO, respectively), as shown in Figures S1–S3. In Na‐ZnO, the unit‐cell volume increased with increasing Na^+^ content until reaching a saturation region that coincided with the maximum ML intensity (Figure S2d). Based on an initial screening of Li and Na substitution levels over the range of 1–10 mol.%, the highest ML intensity was obtained at 1.6 mol.% Li and 3 mol.% Na. Accordingly, these compositions were selected as representative samples for detailed investigation. Scanning electron microscopy (SEM; Figure 1a) revealed that alkali substitution markedly promoted crystal growth, yielding grains of approximately 20 µm together with crater‐like surface textures. These topographies, which have not previously been reported for ZnO, likely originate from defect‐driven volatilization and surface reconstruction promoted by alkali incorporation. Atomic‐resolution scanning transmission electron microscopy (STEM; Figure 1b,c) confirmed the preservation of the wurtzite structure, and fast Fourier transform (FFT) patterns showed (0002) and (10‐10) reflections, indicating long‐range crystallinity. The annular bright‐field (ABF) and high‐angle annular dark‐field (HAADF) images clearly resolved the Zn and O columns. The STEM images confirm that alkali incorporation does not introduce noticeable lattice distortion or secondary phases. Direct visualization of Li^+^, because of its low atomic number, or of dilute Na^+^ in the ZnO lattice, is experimentally challenging using STEM. Therefore, the substitution behavior was verified by synchrotron XRD data (Figures S1–S3), which showed systematic changes in the lattice parameters consistent with Li^+^/Na^+^ occupying Zn^2+^ sites. Raman spectroscopy (Figure 1d) exhibited systematic shifts toward lower wavenumbers compared with ZnO for both Li‐ and Na‐ZnO. This indicates systematic softening of *E*_2_(low) and *E*_2_(high) modes associated with Zn and O sublattice vibrations [30]. These changes are consistent with lattice expansion observed in XRD. In particular, the enhanced *A*_1_(LO) intensity in Na‐ZnO suggests an increased lattice defect concentration [30]. These vibrational signatures support the defect‐dependent optical processes discussed below.

### 2.2 Photoluminescence Properties

**FIGURE 2:**
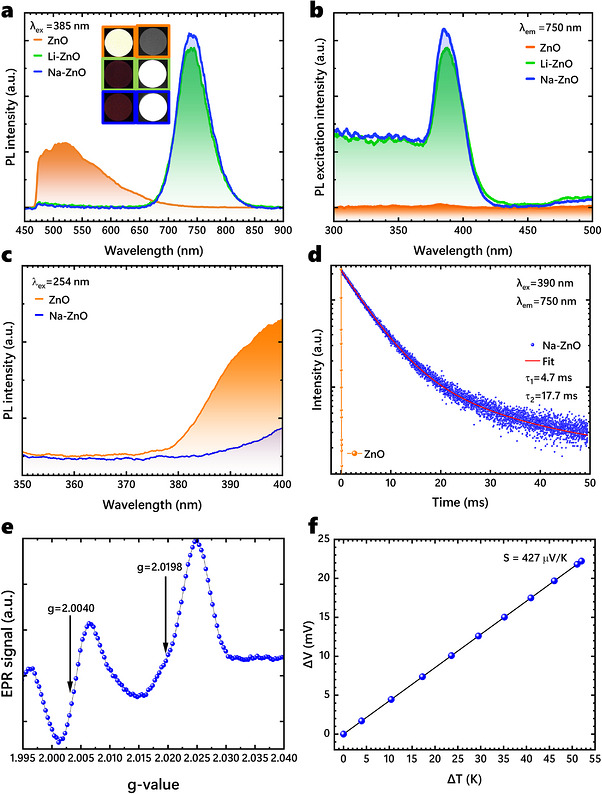
Optical and thermoelectric properties of defect‐engineered ZnO. (a) PL spectra of undoped ZnO, Li‐ZnO, and Na‐ZnO measured at λex = 385 nm. Alkali doping suppresses the visible defect emission of undoped ZnO and gives rise to a red‐to‐near‐infrared (NIR) emission band in the 650–900 nm range, with Na‐ZnO showing the strongest emission centered at ∼750 nm. Inset: photographs of the samples under UV excitation (λex = 365 nm) recorded in the visible (< 700 nm) and NIR (>700 nm) regions. (b) PL excitation spectra monitored at λem = 750 nm, showing the excitation characteristics of the NIR‐emissive states in alkali‐substituted ZnO. (c) Comparison of the near‐band‐edge emission of undoped ZnO and Na‐ZnO under λex = 254 nm, showing the marked suppression of the intrinsic near‐band‐edge emission after Na^+^ doping. (d) PL decay profiles monitored at λem = 750 nm for undoped ZnO and Na‐ZnO, revealing biexponential decay for Na‐ZnO with time constants of τ_1_ = 4.7 ms and τ_2_ = 17.7 ms, consistent with long‐lived deep‐level NIR emission. (e) EPR spectrum of Na‐ZnO, exhibiting distinct paramagnetic signals at *g* = 2.0040 and 2.0198. These values are consistent with previously reported anisotropic EPR signatures of negatively charged zinc‐vacancy‐related defects in the wurtzite ZnO lattice [34, 35, 36]. (f) Thermoelectric characterization of Na‐ZnO, showing a stable and linear ΔT‐ΔV relationship with a positive Seebeck coefficient of S = +427 µV K^−1^, indicating *p*‐type conduction in Na‐ZnO.

As shown in Figure 2a, undoped ZnO exhibits a visible‐band photoluminescence (PL) near 500 nm, which is commonly attributed to intrinsic donor‐related defects such as zinc interstitial (Zn*i*) and oxygen vacancy (Vo) [31, 32]. In contrast, upon partial doping with Li^+^ or Na^+^, this visible emission eventually disappeared. Instead, a broadband red‐to‐NIR PL band (650–900 nm) emerged (Figure S4a–d). The maximum red‐to‐NIR emission was obtained under 385 nm excitation for both optimally doped Li‐ and Na‐ZnO. Among the doped samples, Na‐ZnO shows the most pronounced NIR emission, centered at around 750 nm. The corresponding photographs under UV excitation also confirm the clear appearance of NIR luminescence in the alkali‐doped samples. The PL excitation spectra monitored at λem = 750 nm (Figure 2b) show that the NIR‐emissive states are effectively excited in the near‐UV region, with the strongest response observed λex = 385 nm. This emission at 750 nm (1.65 eV) emission is consistent with a defect‑related transition in ZnO [33]. Importantly, the near‐band‐edge emission of undoped ZnO in the 380–400 nm region is markedly suppressed after Na^+^ doping (Figure 2c), suggesting that the radiative recombination pathways are modified through defect engineering. In other words, Na^+^ incorporation appears to suppress intrinsic band‐edge recombination and instead enhances deep‐level radiative recombination in the NIR region.

This interpretation is further supported by time‐resolved PL measurements. As shown in Figure 2d, the PL decay monitored at an emission wavelength of λem  =  750 nm for Na‐ZnO exhibits biexponential behavior with decay time constants of τ_1_  =  4.7 ms and τ_2_  =  17.7 ms, whereas undoped ZnO shows no detectable emission at this wavelength. These millisecond‐scale decay components are indicative of emission originating from long‐lived deep‐level states rather than near‐band‐edge recombination. Accordingly, Na^+^ doping is considered to modify the defect landscape, leading to a shift of the dominant emission from visible defect‐related luminescence to long‐lived NIR emission.

Consistent with this picture, the electron paramagnetic resonance (EPR) spectrum of Na‐ZnO (Figure 2e) exhibits distinct paramagnetic signals at *g*  =  2.0040 and 2.0198. These values are consistent with previously reported anisotropic EPR signatures of negatively charged zinc‐vacancy‐related defects (denoted as V_Zn_^−1^) in the wurtzite ZnO lattice [34, 35, 36].

To further examine the electronic character of the defect‐engineered ZnO, thermoelectric measurements were performed (Figure 2f; Figure S4e,f). Although Hall measurements are often challenging for ceramic ZnO owing to high carrier concentrations and grain‐boundary effects, the sign of the Seebeck coefficient is commonly used to evaluate the majority carrier type. Na‐ZnO exhibits a stable and nearly linear response, yielding a positive Seebeck coefficient of S  =  +427 µV K^−1^. The voltage polarity was further validated using *p*‐type and *n*‐type Si reference samples measured under identical conditions, indicating that Na‐ZnO exhibits *p*‐type conduction behavior.

Taken together, these optical and thermoelectric results suggest that Na^+^ doping induces *p*‐type transport characteristics while activating long‐lived deep‐level NIR emission in ZnO, resulting in an electronic structure distinct from that of undoped ZnO. Combined PL/ML spectroscopy and density functional theory (DFT) calculations in Section 2.3. and 2.4. further suggest that zinc‐vacancy‐related defects play a dominant role as radiative centers responsible for the 750 nm NIR emission. The stabilization of *p*‐type behavior is therefore considered to facilitate the formation of acceptor‐type defects, which may enhance stress‐induced charge detrapping and NIR ML.

### 2.3 Ultrahigh ML Sensitivity and Stress‐to‐NIR‐Light Conversion in Defect‐Engineered ZnO

**FIGURE 3:**
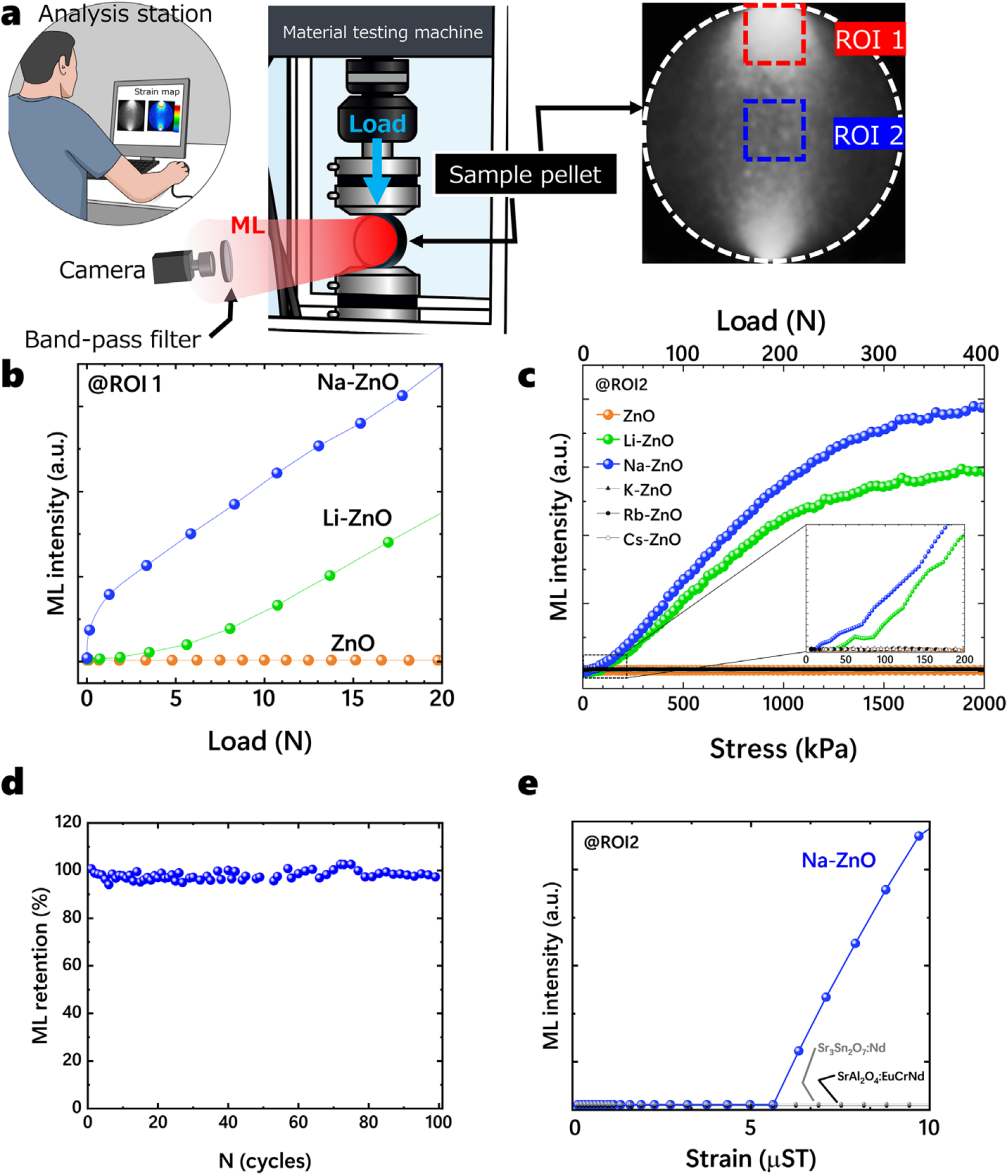
Ultrahigh mechanoluminescence (ML) sensitivity of defect‐engineered ZnO. (a) Schematic of the ML measurement geometry, indicating the two analyzed regions of interest (ROI 1 and ROI 2) on the pellet surface. (b,c) ML intensity profiles collected from ROI 1 and ROI 2, respectively, showing the markedly enhanced response of Na‐ZnO, particularly in ROI 2, where the signal predominantly reflects intrinsic elastic mechanoluminescence (elastic ML). Inset (c): ML responses of K‐, Rb‐, and Cs‐substituted ZnO, showing no measurable emission from ROI 2 under the present conditions. (d) Cyclic elastic ML response of Na‐ZnO (ROI 2) measured over 100 loading cycles, demonstrating excellent repeatability and mechanical robustness. (e) Benchmark comparison of the elastic ML response of Na‐ZnO with previously reported high‐sensitivity ML materials, highlighting the detectable response of Na‐ZnO down to 6 µST in the microstrain regime.

**FIGURE 4:**
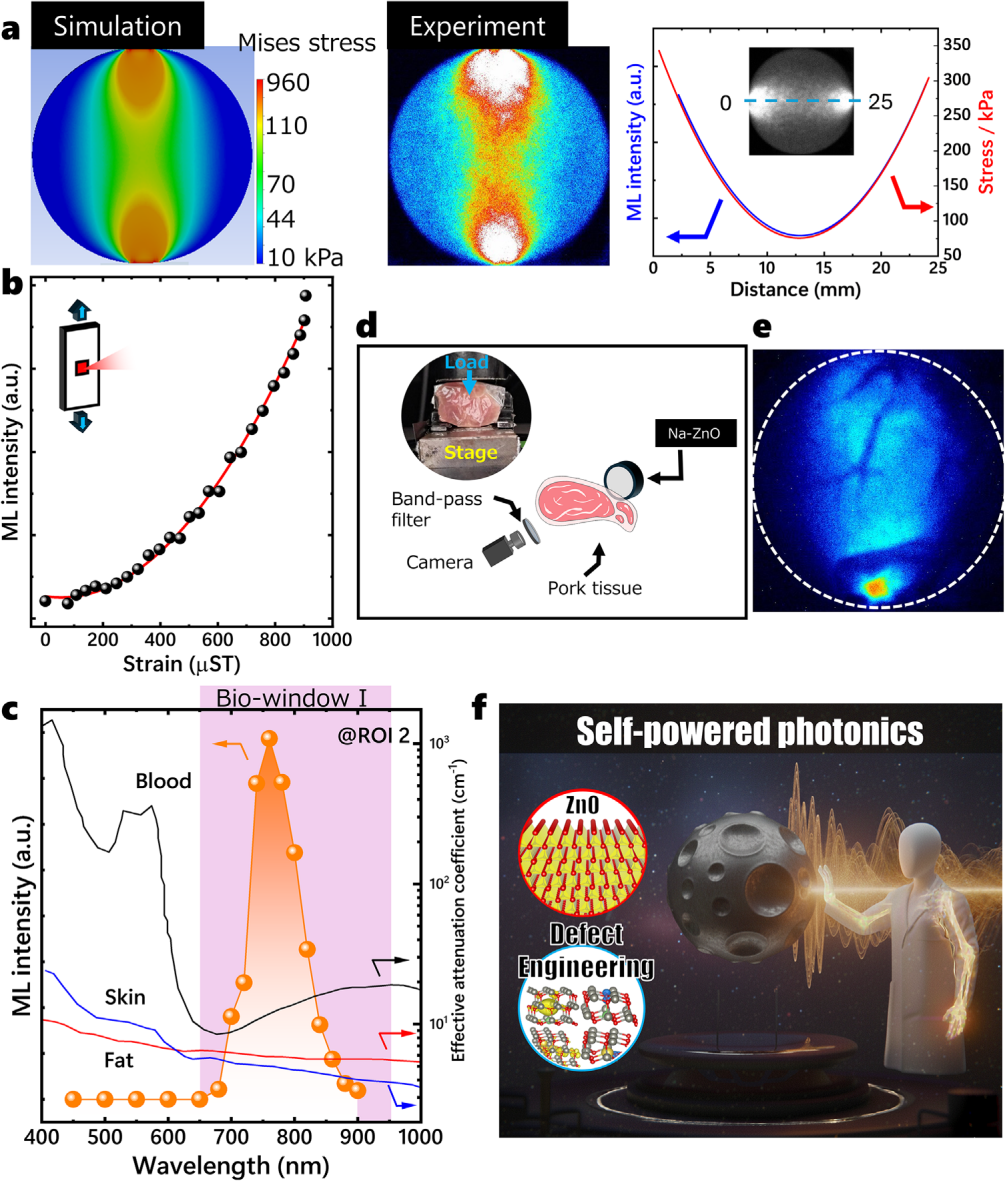
Stress mapping and self‐powered near‐infrared mechanoluminescence (NIR ML) of Na‐ZnO. (a) Finite element method (FEM) simulation of the stress distribution under an applied load of 100 N (left), corresponding experimental ML image of Na‐ZnO (center), and comparison between the simulated stress profile and the measured ML intensity profile across the pellet (right). (b) Tensile‐test ML response of Na‐ZnO in the microstrain regime; inset shows the tensile testing geometry. (c) Experimental setup for tissue‐mediated imaging using stress‐driven NIR ML from Na‐ZnO. (d) Corresponding pseudo‐color ML image recorded through biological tissue with thicknesses ranging from 1 to 7 mm. (e) Band‐pass‐filtered self‐powered ML spectrum of Na‐ZnO, confirming an emission centered at ∼750 nm, consistent with the PL results. (f) Schematic illustration of stress‐to‐NIR‐light conversion in defect‐engineered *p*‐type ZnO, highlighting self‐powered emission within the first biological window (650–900 nm).

Stress‐driven ML was evaluated by quantitative analysis of the light emission during mechanical motion by compression (Figures 3 and 4a), tension (Figure 4b), ultrasound (Figures S5), and friction (Figure S6). Under compressive force, including cyclic ML performance, as shown in Figures 3 and 4, Na‐ZnO exhibited exceptionally strong ML in the kPa range, as revealed by both the simulation (finite element method; FEM) and experimental calibration; the required stresses were six orders of magnitude lower than the reported GPa‐level activation threshold for previous ML materials [37].

As shown in Figure 3a, the ML response was analyzed using two regions of interest (ROI 1 and ROI 2) on the pellet surface to distinguish tribo‐induced and intrinsic elastic ML contributions. The corresponding ML intensity profiles are presented in Figure 3b,c. In both regions, Na‐ZnO exhibited a substantially stronger ML response than undoped ZnO and Li‐ZnO, with the enhancement being most evident in ROI 2. Although K, Rb, and Cs were also examined as potential alkali dopants during preliminary screening, these ions did not show measurable elastic ML as shown in Figure 3c, likely due to their large ionic radii and limited incorporation into the ZnO lattice. Therefore, detailed analysis of these systems is not further included in this study. To evaluate elastic ML, the cyclic stability of Na‐ZnO was evaluated under repeated mechanical loading. As shown in Figure 3d, during a 100‐cycle compression test, the ML intensity exhibited excellent retention with minimal degradation throughout the measurement. This result confirms that the ML response is highly reversible and mechanically robust. A key characteristic of Na‐ZnO is its ultrahigh sensitivity to weak mechanical stimuli. As shown in Figure 3e, the elastic ML response was detectable down to 6 µST, clearly placing the material in the microstrain regime. This sensitivity surpasses that of previously reported high‐sensitivity ML materials, including Sr_3_Sn_2_O_7_:Nd and SrAl_2_O_4_:Eu,Cr,Nd [11, 22], which exhibited no detectable response under identical low‐strain conditions. Moreover, the activation stress of Na‐ZnO lies in the kPa range, corresponding to a reduction of approximately six orders of magnitude relative to the GPa‐level thresholds typically reported for conventional ML materials [37]. These results demonstrate Na‐ZnO as an ultrahigh‐sensitivity ML material capable of converting extremely weak mechanical deformation into a detectable optical signal.

The spatial relationship between mechanical stress and light emission was further examined by combining FEM simulation with experimental ML imaging. Under an applied load of 100 N, the simulated stress distribution closely matched the measured ML intensity profile across the pellet (Figure 4a), indicating that the emitted light directly reflects the local stress field. Consistently, Na‐ZnO also exhibited a clear ML response under tensile deformation in the microstrain regime (Figure 4b), confirming that weak reversible strain can be transduced into an optical signal with high fidelity.

The stress‐driven NIR emission of Na‐ZnO is also attractive for biophotonic applications. Band‐pass‐filtered measurements further confirmed that the ML spectrum is centered at ∼750 nm (Figure 4c), consistent with the PL results. Because this wavelength falls within the first biological window (650–900 nm), where optical absorption by biological media is relatively low, Na‐ZnO is well suited for noninvasive mechano‐optical sensing and biophotonic signaling [6, 7, 8, 9, 10, 11, 12, 13, 14, 15, 16, 17, 18, 19, 20]. To evaluate the penetration capability of the emitted NIR ML, a biological tissue (pork meat) with a thickness of 1 mm was placed on top of the Na‐ZnO sample (Figure 4d). The transmitted NIR ML emission produced high‐contrast images, allowing internal structural differences to be distinguished (Figure 4e). When the tissue thickness was increased to 7 mm, the measured camera intensity at the center region decreased from 39 000 to 18 000 (approximately 80 000 without tissue). Assuming exponential attenuation of the emitted light in tissue, a camera signal of 500 (with a noise level below 100) corresponds to an estimated tissue thickness of 33.9 mm. As shown in Figure 4 and Videos S1 and S2, self‐powered ML images were detectable through both 1 and 7 mm thick pork tissues, indicating sufficient penetration for tissue‐mediated optical readout. The overall concept of stress‐to‐NIR light conversion in defect‐engineered *p*‐type ZnO is illustrated schematically in Figure 4f.

The versatility of alkali‐doped ZnO is further supported by additional stimulation modes. As shown in Figure S5, Na‐ZnO dispersed in water exhibits clear NIR ML under ultrasonic ON/OFF cycling, suggesting that stress‐to‐light conversion can also operate under acoustic stimulation in a liquid environment. In addition, Figure S6 shows that Li‐ZnO generates distinct friction‐induced ML patterns during manual writing under a maximum applied load of 0.8 N (corresponding to a maximum pressure of 20 kPa), highlighting the potential of alkali‐doped ZnO for localized pressure visualization and interactive mechano‐optical readout.

These observations are notable because single‐phase ZnO has generally not been regarded as an ML material. In contrast, previously reported ZnO‐based ML systems have typically relied on extrinsic activators, such as Mn^2+^, and emitted visible light around 585 nm from multiphase heterostructures [38]. In contrast to these previous reports, the present work shows that simple alkali doping can transform ZnO into a defect‐engineered *p*‐type semiconductor exhibiting self‐powered NIR ML with high sensitivity to weak mechanical stimuli.

### 2.4 Deep‐Level Defects and ML Mechanism

**FIGURE 5:**
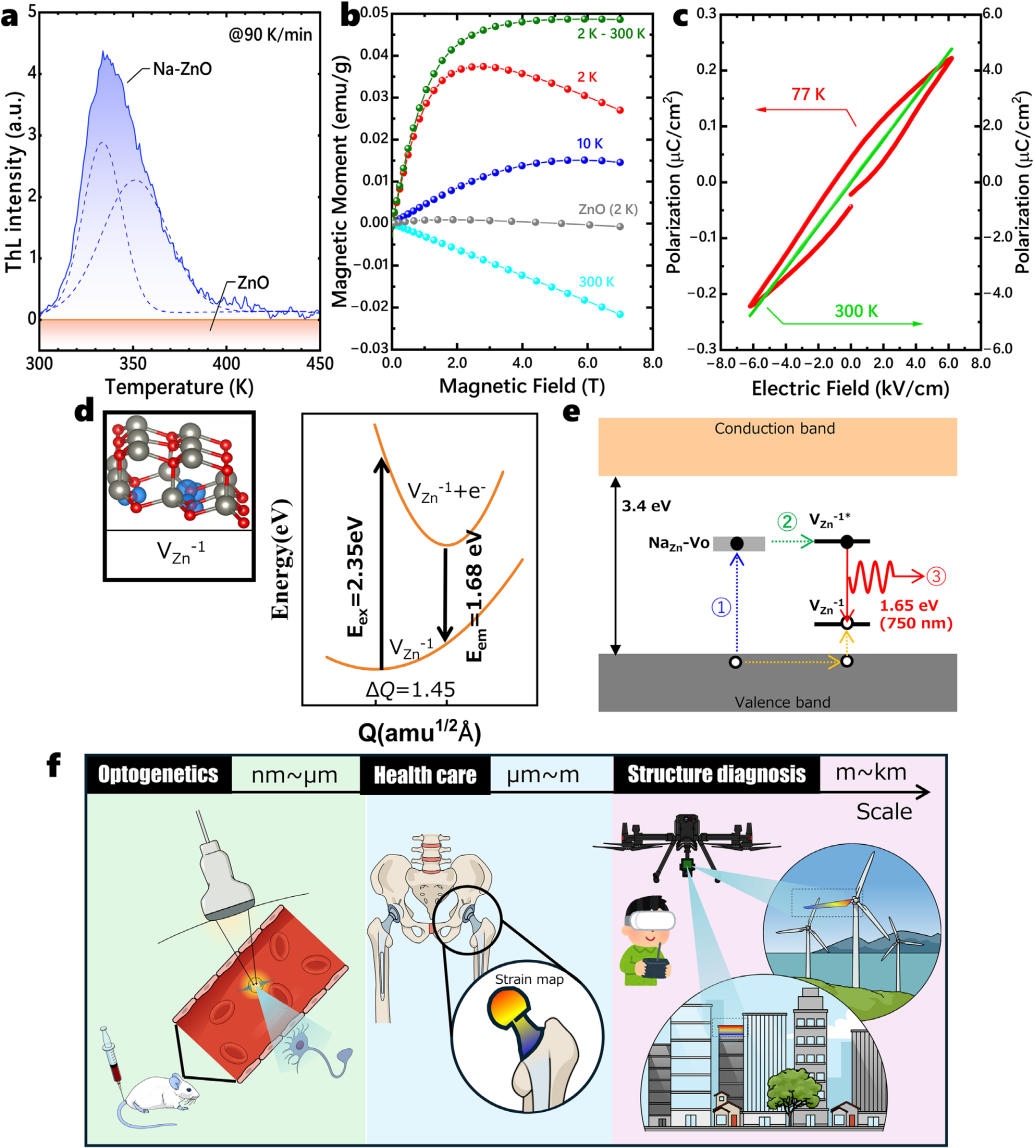
Deep‐level defects and proposed mechanism of self‐powered near‐infrared mechanoluminescence (NIR ML) in defect‐engineered ZnO. (a) Thermoluminescence (ThL) glow curves of undoped ZnO and Na‐ZnO, with peak fitting of Na‐ZnO indicating trap depths of approximately 1.15 and 1.36 eV. (b) Magnetization curves of undoped ZnO at 2 K and Na‐ZnO measured at different temperatures. (c) Polarization‐electric field (*P‐E*) hysteresis loops of Na‐ZnO measured at 77 and 300 K. (d) Density functional theory (DFT) and configurational‐coordinate analysis of the Zn vacancy defect, showing an emission energy of 1.68 eV. (e) Schematic illustration of the proposed self‐powered NIR ML mechanism involving deep – trap states and ‐related radiative centers. (f) Conceptual illustration of multiscale mechano‐optical applications enabled by defect‐engineered ZnO.

Alkali doping introduces deep‐level defects into ZnO. Thermoluminescence (ThL) measurements revealed two prominent trap depths in Na‐ZnO, whereas undoped ZnO exhibits no detectable traps (Figure 5a; Figure S7). The deep traps located at approximately 1.15 and 1.36 eV persisted at room‐temperature, indicating that they serve as robust reservoirs for stress‐to‐light emission. The defect levels were evaluated and classified through a combination of PL (Figure 2) and ThL (Figure 5a; Figure S7) measurements, which enable differentiation between shallow donor‐related transitions and deep acceptor‐type states associated with V_Zn_‐related complexes. In addition, DFT calculations were performed to estimate the charge‐state transition levels of Na_Zn_, V_Zn_, and their defect complexes. The experimentally observed PL/ThL peaks showed excellent agreement with the calculated transition energies, supporting the assignments of the corresponding defect levels.

The trapped carriers have brought out unexpected magnetic and ferroelectric properties. At 2 K, Na‐ZnO showed a saturated *M‐H* component superimposed on the diamagnetic curve of unsubstituted ZnO (Figure 5b). Since no magnetic sources exist in the Na‐ZnO, the saturated *M‐H* component is apparently produced by the trapped carriers. Notably, coupling trapped carriers and ferromagnetic behaviors could be obtained with enhanced defect concentrations in ZnO, like in the typical ML material SrAl_2_O_4_:Eu^2+^ [39]. More strikingly, acceptor‑type defects have given rise to ferroelectric *P‐E* hysteresis (Figure 5c), which is consistent with a previous report [40]. Generally, pure wurtzite ZnO has been known as non‐ferroelectric [41]. Our findings mark defect‑engineered ZnO as the most chemically and structurally simple ML material (Table S1) by coupled electronic and structural effects in the ferroelectric state.

DFT calculations further identified Zn vacancies as the only defects capable of reproducing the 1.68 eV (738 nm) emission predicted by a configurational‐coordinate (CC) model (Figure 5d). The calculations of charge‐state transition levels in Na‐ZnO further revealed that the Na_Zn_‐V_o_ coupled defect state lies approximately 1.3 eV below the conduction band, functioning as an effective carrier trap (Figure S8a,b).

Together, these results support the following self‐powered emission (Figure 5e):

1. Carriers are trapped in the deep defect states associated with Na_Zn_‐Vo.
2. Mechanical strain induced charge transfer from Na_Zn_‐Vo to the excited V_Zn_^−1^* states.
3. The released holes recombine with the excited V_Zn_^−1^* states, resulting in the emission of 750 nm photons.

The ThL experimental and DFT theoretical investigation for Li‐ZnO showed similar electronic structures and defect levels as Na‐ZnO, indicating a common underlying mechanism for stress‐to‐light emission.

The activation of ML in this work is primarily attributed to the synergistic coupling between defect‐induced ferroelectricity and high‐efficiency carrier dynamics. While native wurtzite ZnO is generally considered non‐ferroelectric, the alkali‐doping strategy introduces acceptor‐type defects that give rise to distinct ferroelectric *P‐E* hysteresis. This newly emergent ferroelectric state allows the material to generate significant internal electric fields even under minimal elastic strain, which effectively lowers the energy barrier required to trigger charge transfer from deep‐level traps. Specifically, the mechanical strain induces a highly efficient transfer of electrons from the stable Na_Zn_‐V_o_ coupled defect states to the excited V_Zn_ states. Besides, Alkali doping significantly enhances grain growth in ZnO. Larger grain sizes reduce the number of grain boundaries, thereby minimizing light scattering and non‐radiative recombination at interfaces, which improves NIR emission efficiency. Moreover, the characteristic crater‐like surface morphology observed in Li/Na‐ZnO may act as a local stress concentrator, generating enhanced strain gradients even under low macroscopic loads and thus contributing to the high ML sensitivity.

## 3 Conclusion

The discovery of strong stress‐to‐light conversion, specifically elastic ML, in defect‐engineered ZnO expands the fundamental framework of ML research by showing that lattice defects in a chemically and structurally simple oxide lattice can serve as active photon emitters (Table S1). By stabilizing *p*‐type conduction through controlled defect engineering, this work overcomes the inherent *n*‐type character of ZnO and activates a new radiative pathway that yields intense NIR emission (650–900 nm; bio‐window I) with high sensitivity and measurable tissue penetration (Figure 4d,e; Videos S1, and S2). This self‐powered emission is driven solely by endogenous mechanical stimuli such as ultrasound (Figure S5), eliminating the need for external electrical power and wiring.

This advance offers scalable, rare‐earth‐free solutions for biophotonics, healthcare, and infrastructure monitoring, with global implications for sustainable sensing and energy technologies. Here, scalability refers to both multiscale applicability and practical manufacturability. As illustrated in Figure 5f, Li/Na‐doped ZnO enables stress sensing across a wide range of length scales, from nanometer‐scale optical interrogation to large‐scale structural health monitoring, owing to its single‐phase wurtzite structure with controlled intrinsic defects. In addition, the material can be synthesized from earth‐abundant and air‐stable oxide precursors without complex processing, supporting large‐volume and low‐cost production.

These capabilities open pathways for implantable health monitors, biophotonic communication tools, and smart infrastructure sensors, creating light without electricity and enabling real‐time stress visualization at multiple scales (Figure 5f). Furthermore, ZnO's earth‐abundant, non‐toxic, and recyclable nature distinguishes it from rare‐earth‐based ML materials and offers a sustainable platform for high‐performance NIR photonics.

## 4 Experimental Section

### 4.1 Sample Preparation

To control defects in ZnO crystal, high‐purity powders were chosen as starting materials. Alkali dopants (Li_2_CO_3_, Na_2_CO_3_, K_2_CO_3_, Rb_2_CO_3_, Cs_2_CO_3_; ≥ 99.9%, Kojundo Chemical Lab., Japan) were weighed to target molar ratios of Li: 0%–10%, Na: 0%–10%, and K/Rb/Cs: 0%–5%. Mixtures were homogenized in an agate mortar and calcined in air/Ar/N_2_/O_2_/H_2_‐Ar at 1173–1573 K for 1–20 h. To control the formation of the defects, the calcination conditions, including atmosphere, temperature, and duration, were systematically varied. Oxidizing conditions promote the formation of zinc vacancies (V_Zn_), which act as deep acceptor centers responsible for the 750 nm NIR emission. In contrast, inert or reducing atmospheres tend to suppress V_Zn_ formation and favor donor‐like oxygen vacancies (V_O_), thereby diminishing the desired NIR ML response. Among the tested conditions, calcination in air at 1373 K for 1 h yielded the optimal concentration of V_Zn_‐related defects for efficient ML in Na‐ZnO.

For ML measurements, test pellets (diameter, 25 mm; thickness, 15 mm) composed of each defect‐engineered powder sample and epoxy resin were prepared. The test pellet used for ML measurements was fabricated using a two‐layer configuration. The active layer was prepared by mixing 0.5 g of ZnO powder with 0.5 g of epoxy resin (50 wt.%). This layer was then backed with a second layer consisting of 4.5 g of pure epoxy resin to ensure mechanical stability and uniform load transfer during testing. Ceramic disks were fabricated by cold isostatic pressing at 180 MPa, followed by sintering (relative density 90%). Bars (1 × 1 × 2 mm^3^) were cut from the ceramics pellets and fitted with Ag electrodes for thermoelectric measurements. For ferroelectric tests, pellets were polished to 1.45 mm thickness and 15.5 mm diameter, and Au electrodes were deposited on both faces.

### 4.2 Structural Characterization

X‐ray diffraction (XRD) was carried out using synchrotron X‐rays (λ = 0.496037 Å) at BL02B2 (SPring‐8, Japan). Rietveld refinement (GSAS) quantified phase composition, dopant substitution levels, defects, and lattice parameters. Raman spectroscopy was conducted using a 532 nm laser (NRS‐5100, JASCO Corporation, Japan). Scanning electron microscopy (SEM) was performed at 20 kV to observe the grain size and morphology (JSM‐IT800, JEOL Ltd., Japan). Scanning transmission electron microscopy (STEM) was conducted in both the HAADF, LAADF, and ABF modes (ARM‐200F, JEOL Ltd., Japan).

### 4.3 Optical Measurements

PL and PL excitation spectra were measured using a spectrofluorometer equipped with an Xe lamp (FP‐6600, JASCO Corporation, Japan). NIR fluorescence images were captured using a charge‐coupled device (CCD) camera (VLU‐12M, Baumer, Germany) with long‐pass filters (> 700 nm) to isolate NIR emissions. PL lifetime measurements were performed using a fluorescence lifetime spectrometer (Quantaurus‐Tau C11367‐05, Hamamatsu Photonics K.K.). The PL decay curves were recorded using the time‐correlated single photon counting (TCSPC) method. An Xe‐lamp equipped with 390 nm band‐pass filter (FWHM: 10 nm) was used as the excitation light source. The emission was monitored at 750 nm, corresponding to the peak position of the PL spectrum. The obtained decay curves were analyzed by a bi‐exponential fitting function.

### 4.4 ML Measurements

ML measurements were performed using a lab‐built ML measurement system integrated with a material testing system and a photo measurement system, including cameras and photomultiplier tubes. Briefly, the pellet was placed on the stage, and compressive loads from 0–400 N were applied at a rate of 3 mm/min. The strain at the center of the pellet was estimated based on a calibration using a commercial strain gage (Kyowa Electronic Instruments Co., Ltd., Tokyo, Japan). Emission images were recorded using a CCD camera (VCXU‐15M, Baumer, Germany; Figure 3a; Video S3). Regions of interest (ROI 1,2) were used to quantify the light emission at the contact interfaces and the central region. For the ML spectrum, band‐pass filters with a 10 nm full width at half maximum (FWHM) were inserted in front of the camera, and the ML intensity was recorded using different filters with various central wavelengths. The tensile ML test was performed as follows: The powder particles were fixed onto an Al substrate (0.3 mm) equipped with a strain gauge. Tensile strain was applied using a mechanical testing machine (ISL‐500, SANKO Co., Ltd.), and the emitted light was evaluated using a photomultiplier tube. For ML by ultrasound, 1 g of Na‐ZnO powder was dispersed in 20 mL of ultrapure water and stirred in an ultrasonic bath. The emission was recorded with a CCD camera using a band‐pass filter (Figure S5a).

### 4.5 ThL, Magnetic and EPR Measurements

ThL was measured using a heating stage from 150–400 K at a heating rate of 10, 30, 50, 70, 90 K/s (TP94, Linkam Scientific Instruments, United Kingdom). The emission intensity was recorded using a spectrofluorometer (FP‐8600, JASCO Corporation, Japan). Trap depths were estimated using peak analysis based on the Hoogenstratten method. Magnetization measurements were conducted using a superconducting quantum interference device (SQUID) magnetometer at 2–300 K. EPR spectra were acquired using an X‐band ESR spectrometer (X330, JEOL Ltd., Japan) at room temperature. The microwave frequency was 9.151 GHz, and the microwave power was set to 0.998 mW. The magnetic field was swept from 30.0 to 530.0 mT. Field modulation was applied at a frequency of 100 kHz with a modulation width of 0.5 mT. All measurements were performed at room temperature.

### 4.6 Thermoelectric Measurements

The ceramic bar was fixed between AlN substrates, and the thermoelectric voltage was measured using an electrometer (6517A, Keithley Instruments, United States) by heating one side of the substrate with a soldering iron (Figure S4e). Single‐crystal silicon substrates of *p*‐type Si (100) and *n*‐type Si (111) were used as reference samples (0.5 × 0.5 × 2 mm^3^).

### 4.7 Ferroelectric Measurements

The *P‐E* hysteresis loops (10 Hz, Sine wave) were tested by a ferroelectric test unit (PK‐CPE23A‐AC, PolyK Technologies, LLC., United States).

### 4.8 Computational Methods

First‐principles calculations were performed within the framework of density functional theory (DFT) using the Vienna ab initio Simulation Package (VASP). The Perdew–Burke–Ernzerhof (PBE) functional within the generalized gradient approximation (GGA) was employed to describe the exchange‐correlation interactions. To properly account for the strong correlation effects among the localized outer shell electrons in the element, the PBE+*U* approach was adopted, where the on‐site Coulomb correction parameters were set to U(Zn‐3*d*) = 10 eV and U(O‐2*p*) = 7 eV, consistent with values commonly used in previous studies of ZnO systems. The projector augmented‐wave (PAW) method was used to describe the electron‐ion interactions. To model an experimental doping concentration of approximately 1%, a 4 × 4 × 2 ZnO supercell based on the wurtzite structure was constructed, where one Zn atom was substituted with Li or Na to simulate the defect. All structures were fully relaxed until the Hellmann–Feynman forces on each atom were less than 0.005 eV Å^−1^, and the energy convergence criterion was set to 10^−^^7^ eV. A kinetic energy cutoff of 550 eV was used for the plane‐wave basis, and a Gaussian smearing width of 0.02 eV was applied. Only the Γ‐point was considered for Brillouin zone sampling during geometry optimization and total energy calculations. The formation energies and thermodynamic charge‐state transition levels of point defects were calculated following the well‐established formalism [42]. The formation energy of a defect *X^q^* in charge state *q* is expressed as:

$$\begin{array}{ccc} E^{f}\left[X^{q};\varepsilon_{F}\right] & = & E_{\mathrm{tot}}\left[X^{q}\right]-E_{\mathrm{tot}}\left[\mathrm{bulk}\right]\\ & & -\sum_{i}\;n_{i}\mu_{i}+q\left(\varepsilon_{F}+\varepsilon_{\mathrm{VBM}}\right)+{E_{\mathrm{corr}}}^{\mathrm{eFNV}}\left(q\right) \end{array}$$

where *E_tot_* [*X^q^*] and *E_tot_* [*bulk*] represent the total energies of the defect‐containing and perfect ZnO supercells, respectively. *n_i_* and *µ_i_* denote the number and chemical potential of species *i* added to or removed from the system, and *ε*_F_ is the Fermi level referenced to the valence‐band maximum (VBM, *ε*_VBM_). For charged defects, the Freysoldt‐Neugebauer‐Van de Walle (FNV) correction scheme was applied to eliminate spurious electrostatic interactions between periodic images. All defect formation energies were calculated under O‐rich conditions, corresponding to the experimental synthesis environment where the ZnO samples were prepared in ambient air. Under these conditions, the chemical potential of oxygen (*µ_o_*) is maximized and defined as half the total energy of an O_2_ molecule, while the chemical potential of zinc (*µ_Zn_*) is determined from the thermodynamic equilibrium of ZnO:

$$\Delta H_{f}\left(\mathrm{ZnO}\right)=\mu_{o}+\mu_{\mathrm{Zn}}$$

where Δ*H_f_* (ZnO) is the formation enthalpy of ZnO. The chemical potentials of Na dopants were referenced to Na_2_O, according to

$$\mu N_{a}=\left[E_{\mathrm{tot}}\left(Na_{2}O\right)-\mu_{o}\right]/2$$

This choice ensured thermodynamic consistency with the O‐rich synthesis conditions and accurately represented the stability limits of the doped ZnO system. The thermodynamic charge‐state transition levels and optical transition energies of the defects were evaluated based on the total energy differences obtained from first‐principles calculations. The charge‐state transition level ε(*X^q^_1_* | *X^q^_2_*) between charge states *q_2_* and *q_1_* is defined as the Fermi level position where the formation energies of the two charge states are equal.

$$\varepsilon \left({X^{q}}_{1}|{X^{q}}_{2}\right)=\left(E^{f}\left[{X^{q}}_{1};0\right]-E^{f}\left[{X^{q}}_{2};0\right]\right)/\left(q_{2}-q_{1}\right)$$

The optical transition energy was estimated to use a simplified configuration coordinate (CC) model [43]. considering only the key defect geometry associated with the luminescent transition. This energy corresponds to the vertical excitation between the ground and excited electronic states of the defect. Because the structural relaxation of both states was not extensively sampled, the calculated results represent a qualitative description of the defect‐related emission process.

## Author Contributions

C.N.X. conceived and supervised the project. K.O., R.O., and T.U. prepared samples and performed optical measurements. X.G.Z. carried out magnetization measurements. E.N. conducted X‐ray diffraction. G.Y. and Y.C. performed DFT calculations. X.G.Z investigated ferroelectricity. T.U. and K.O. drafted the manuscript; X.G.Z. and C.N.X. provided substantial revisions.

## Conflicts of Interest

The authors declare no conflicts of interest.

## Supporting information




**Supporting File 1**: advs75587‐sup‐0001‐SuppMat.docx.

**Supporting File 2**: advs75587‐sup‐0002‐VideoS1.mov.

**Supporting File 3**: advs75587‐sup‐0003‐VideoS2.mov.

**Supporting File 4**: advs75587‐sup‐0004‐VideoS3.mov.

## Data Availability

The data that support the findings of this study are available from the corresponding author upon reasonable request.
