# Supplementary material for: Stress‐to‐Light Conversion in an Earth‐Abundant Oxide Semiconductor
Source: Adv Sci (Weinh). 2026 May 8;13(43):e75587. doi: 10.1002/advs.75587 (PMC13335826; doi:10.1002/advs.75587)
Supplement: Supplementary file 1 — Supporting File 1: advs75587‐sup‐0001‐SuppMat.docx. [file ADVS-13-e75587-s004.docx]

**Supporting Information**

**Stress-to-light conversion in an earth-abundant oxide semiconductor**

*Tomoki Uchiyama^1,2^, Koki Otonari^2^, Reona Omori^2^, Guangfa Yang^2^, Eiji Nishibori^3^, Ying Chen^4^, Xu-Guang Zheng^,1,5^, Chao-Nan Xu^1,2^**

*Corresponding author: chao-nan.xu.c8@tohoku.ac.jp

T.U. and K.O. contributed equally to this work.

T. Uchiyama, X.G. Zheng, C.N. Xu

^1^ Department of Material Science and Engineering, Faculty of Engineering, Tohoku University, Sendai, Miyagi, 980-8579, Japan

T. Uchiyama, K. Otonari, R. Omori, G. Yang, X.G. Zheng, C.N. Xu

^2^ Department of Material Science and Engineering, Graduate School of Engineering, Tohoku University, Sendai, Miyagi, 980-8579, Japan

E. Nishibori

^3^ Department of Physics, Faculty of Pure and Applied Sciences, Tsukuba Research Center for Energy Materials Science, Hydrogen Boride Research Center (HBRC), and Tsukuba Institute for Advanced Research (TIAR), University of Tsukuba, Ibaraki, 305-8571 Japan

Y. Chen

^4^ Global Learning Center, Tohoku University, Sendai, Miyagi, 980-8579, Japan

X.G. Zheng

^5^ Department of Physics, Faculty of Science and Engineering, Saga University, Saga, 840-8502, Japan

Abbreviations used in this Supporting Information are defined at their first appearance.

**
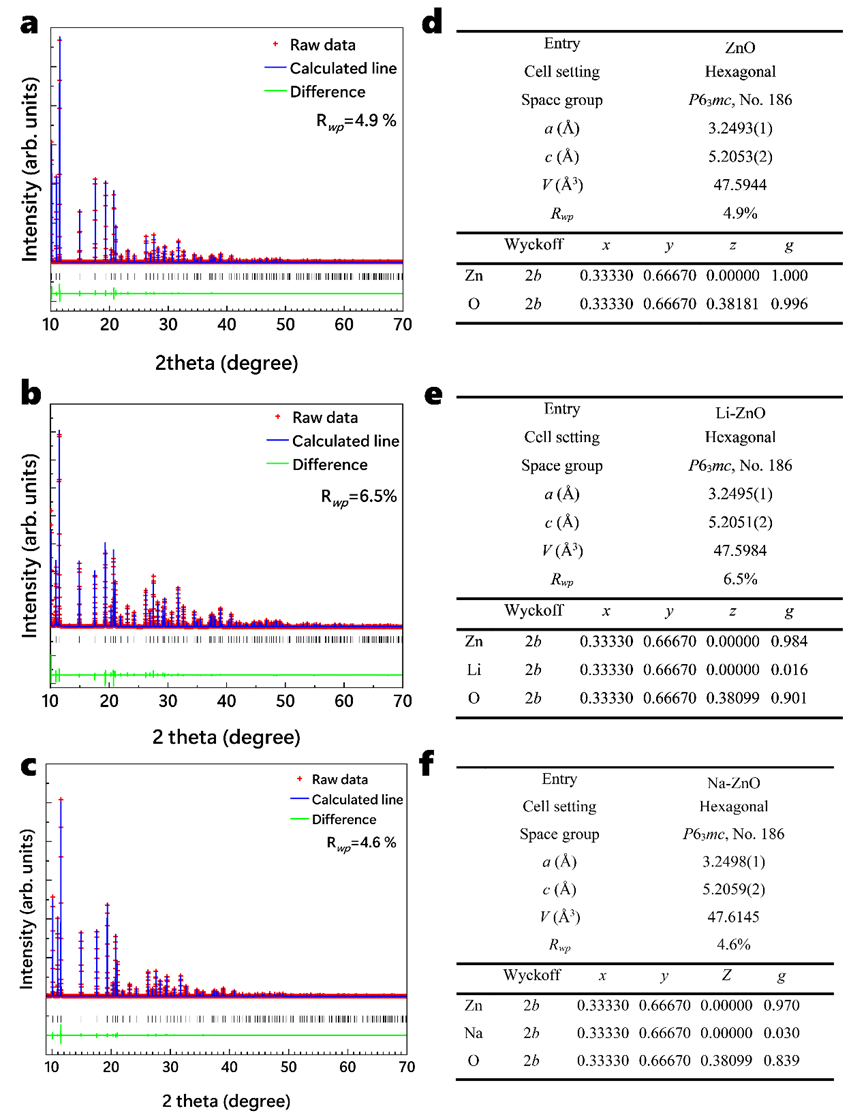
**

**Figure S1. XRD patterns and structural parameters of representative Li- and Na-doped ZnO.**

X-ray diffraction patterns of (a) undoped ZnO, (b) 1.6 mol% Li‑doped ZnO (Li-ZnO), and (c) 3 mol% Na‑doped ZnO (Na-ZnO), together with the corresponding structural parameters (d-f) obtained by Rietveld refinement (λ = 0.496037 Å).

**
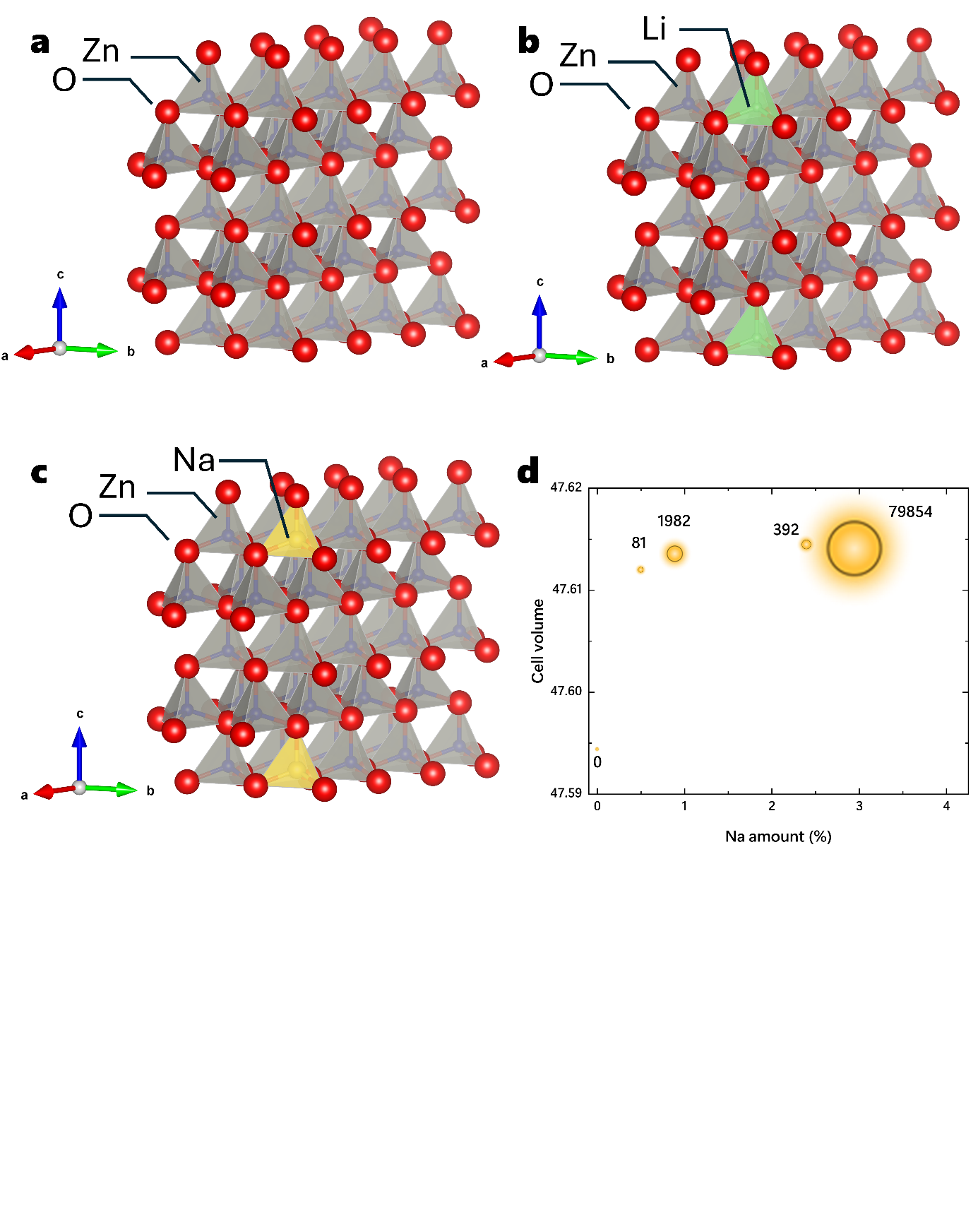
**

**Figure S2. Crystal structures and correlation between unit‑cell volume and stress‑driven mechanoluminescence (ML) intensity in defect‑engineered ZnO.**

Crystal structures of **(a)** undoped ZnO, **(b)** 1.6 mol%Li‑doped ZnO (Li-ZnO), and **(c)** 3 mol%Na‑doped ZnO (Na-ZnO). **(d)** Dependence of the unit‑cell volume and ML intensity on the Na doping concentration in Na-ZnO, showing a maximum at 3 mol% doping. The unit‑cell volume increased with Na^+^ content until reaching a saturation range that coincided with the maximum ML intensity.

**
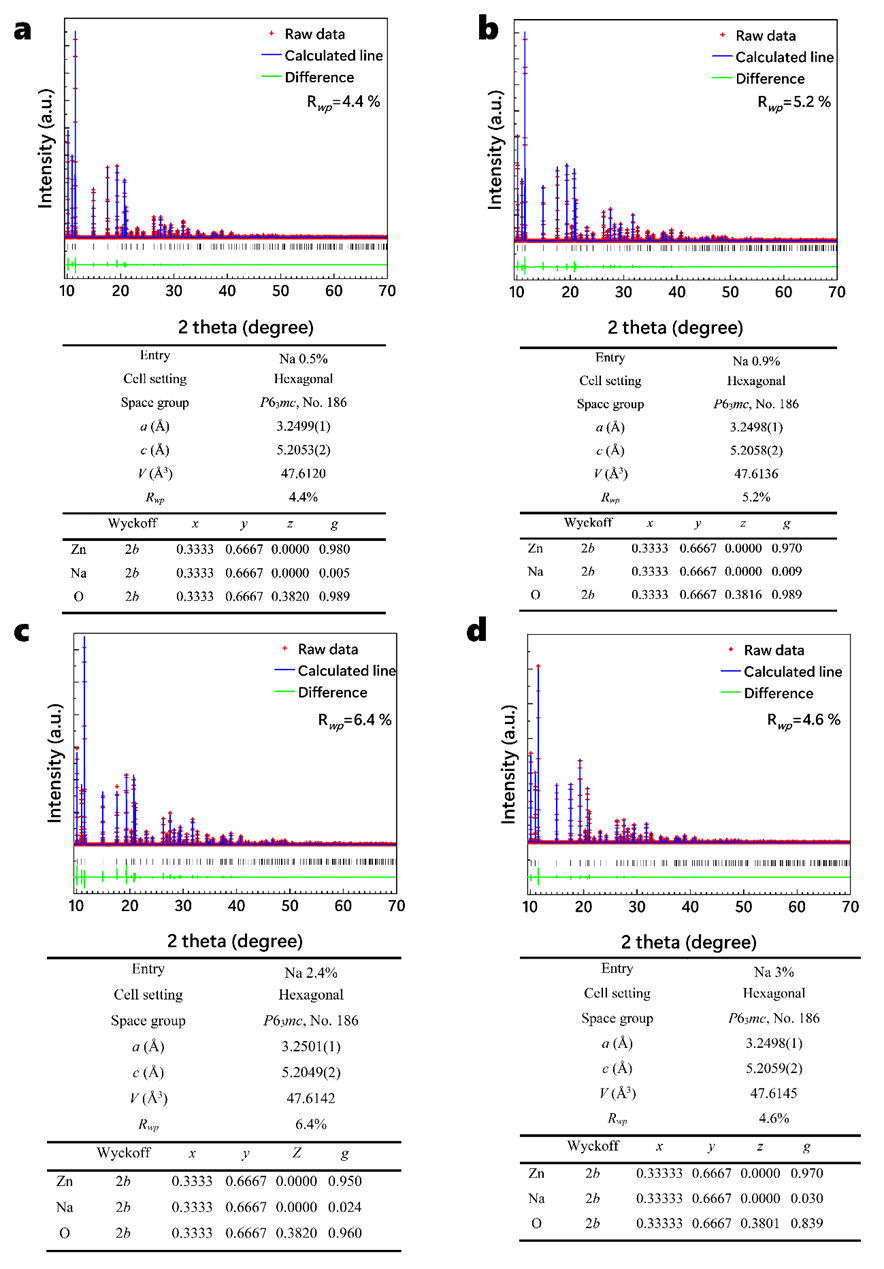
**

**Figure S3. XRD patterns and structural parameters of Na-ZnO with different doping levels.**

XRD patterns and corresponding structural parameters of Na-ZnO with Na doping levels of **(a)** 0.5 mol%, **(b)** 0.9 mol%, **(c)** 2.4 mol%, and **(d)** 3 mol%, obtained by Rietveld refinement (λ = 0.496037 Å).

**
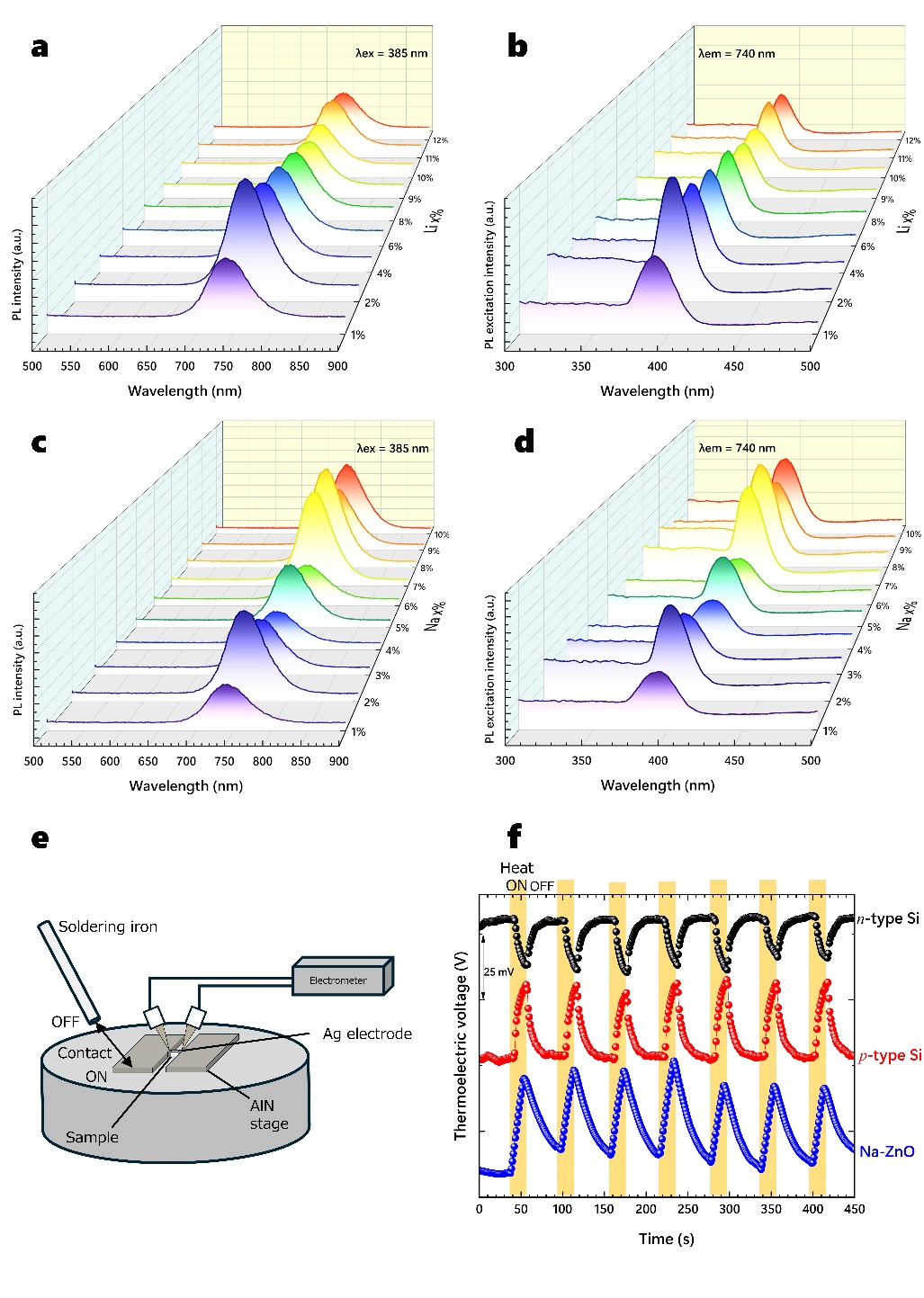
**

**Figure S4. Photoluminescence and thermoelectric properties of Li‑ and Na‑doped ZnO.**

**(a)** PL and **(b)** PL excitation spectra of Li-ZnO. **(c)** PL and **(d)** PL excitation spectra of Na-ZnO. PL spectra were measured at λex = 385 nm and PL excitation spectra were monitored at λem = 750 nm. Alkali substitution suppresses the visible defect emission of undoped ZnO and induces a red-to-near-infrared (NIR) emission band in the 650-900 nm range, with Na-ZnO showing a dominant emission centered at ~750 nm. **(e)** Experimental setup for thermoelectric voltage measurements. The ceramic bar was fixed on between AlN substrates, and the thermoelectric voltage was measured using an electrometer by heating one side of the substrate with a soldering iron. **(f)** Thermoelectric voltage response under heat ON/OFF cycles for *n*‑type Si (black), *p*‑type Si (red), and Na-ZnO (blue). Na-ZnO showed a positive Seebeck coefficient of *S* = +427 μV K^-1^. The voltage polarity was calibrated using *p*-type and *n*-type Si reference samples measured under identical conditions. These results identify Na-ZnO as a *p*-type semiconductor by thermoelectric measurements.

**
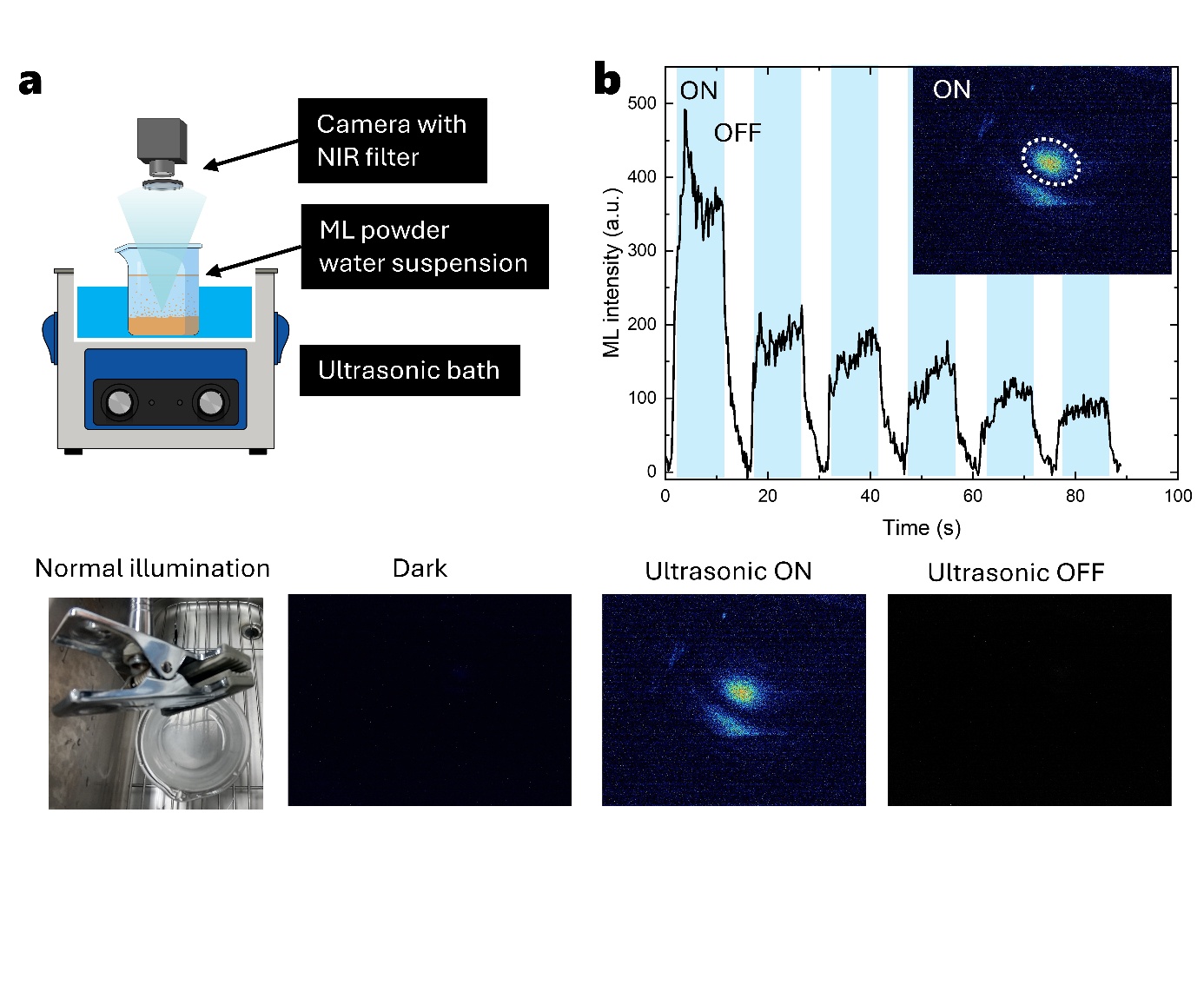
**

**Figure S5. Near infrared mechanoluminescence (NIR ML) by ultrasonic stimulation.**

Na-ZnO powder dispersed in ultrapure water exhibiting NIR ML under ultrasonic stimulation.

**(a)** Experimental set-up. 1 g of Na-ZnO powder was dispersed in 20 mL of ultrapure water and stimulated in an ultrasonic bath. The emission was recorded with CCD camera using a band-pass filter. (**b)** NIR ML response following ON/OFF cycles. Inset: Pseudo-color images of NIR ML under normal illumination, dark conditions, and during ultrasonic ON and OFF states. Na-ZnO dispersed in water exhibited clear NIR ML under ultrasonic ON/OFF cycling, indicating that the stress-to-light conversion can also operate under acoustic stimulation in a liquid environment.

**
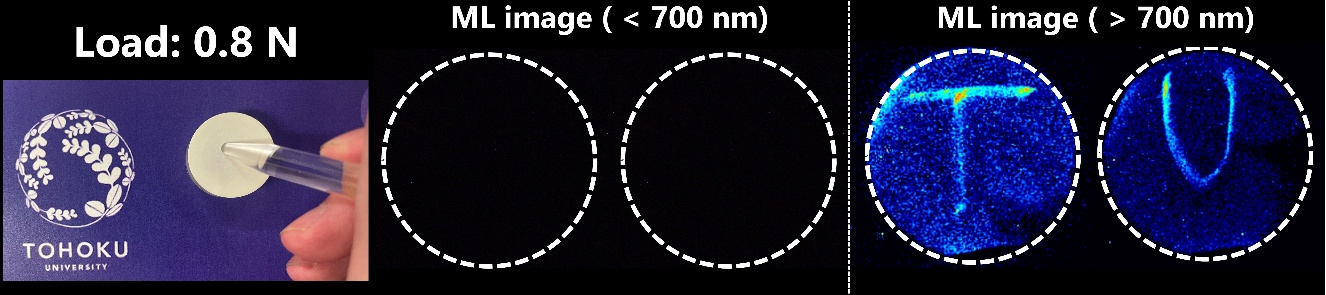
**

**Figure S6. NIR ML image of Li-ZnO under friction.**

ML images obtained when the letters “T” and “U” were written onto the test pellet by applying maximum press load 0.8 N (max pressure 20 kPa, contact area 40 mm^2^), highlighting the potential of alkali-substituted ZnO for localized pressure visualization and interactive mechano-optical readout.

**
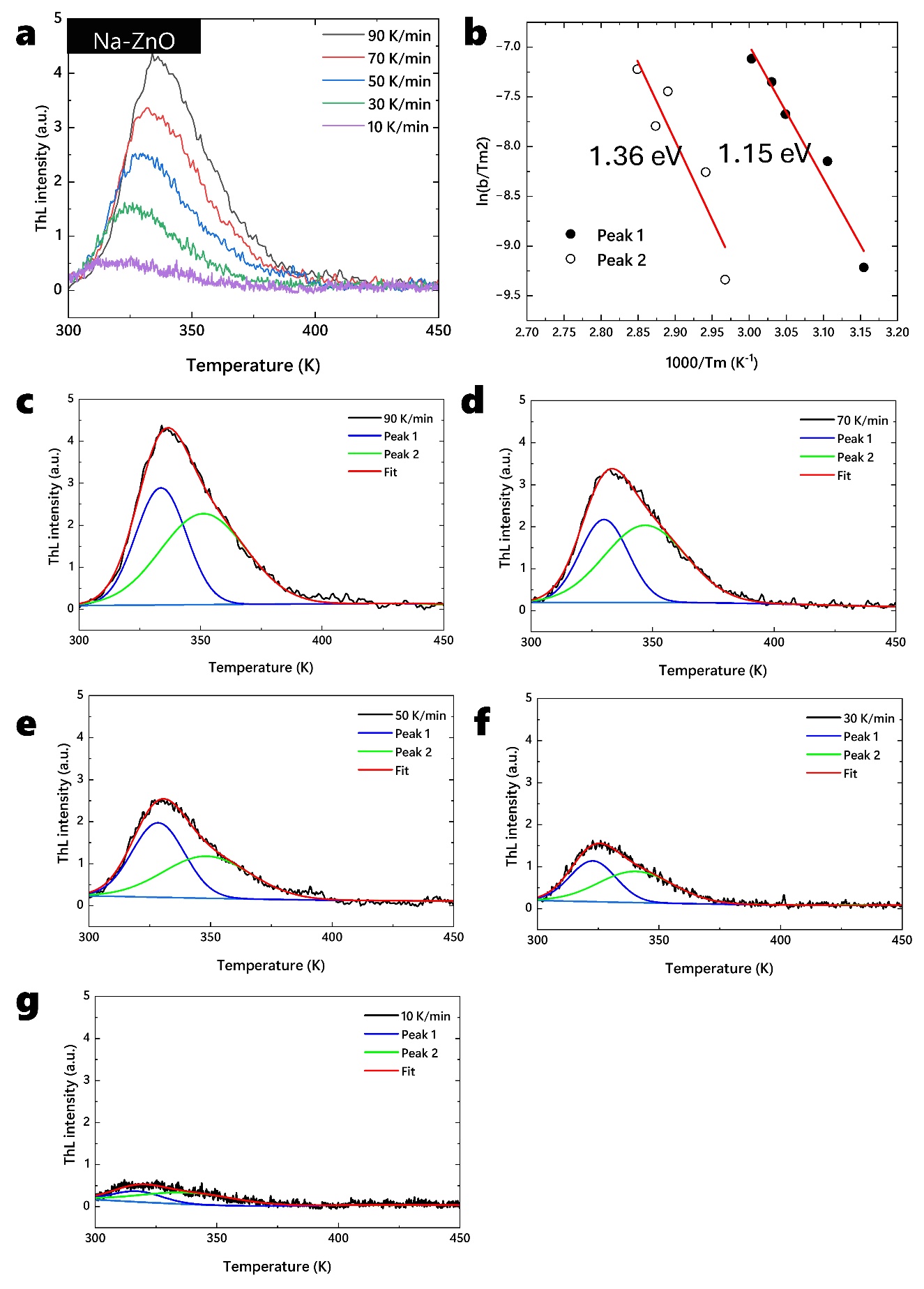
**

**Figure S7. Thermoluminescence (ThL) analysis of Na-ZnO.**

**(a)** ThL glow curves at different heating rates. (**b)** Trap-depth analysis by Hoogenstraaten’s method. (**c-g)** Peak deconvolution of ThL glow curves at heating rates of 90, 70, 50, 30, and 10 Kmin^-1^. The deep traps located at approximately 1.15 and 1.36 eV persisted at room temperature, indicating that they serve as robust reservoirs for stress-to-light emission.

**
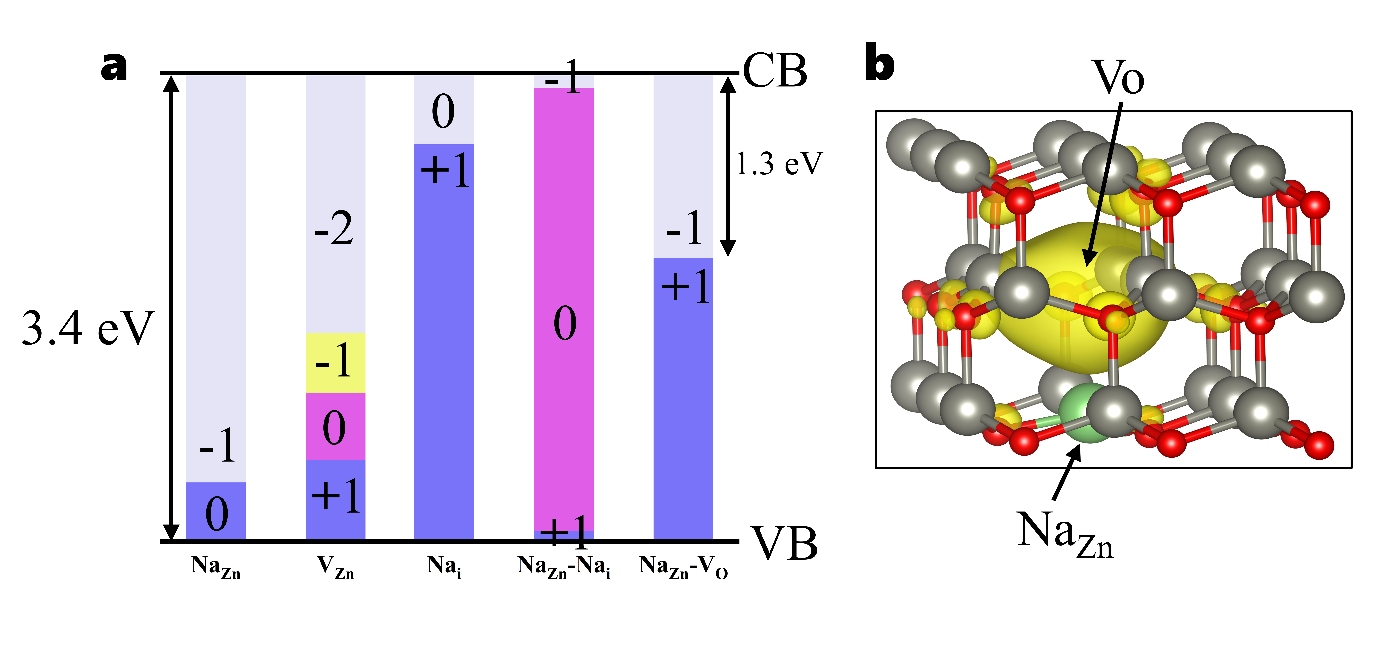
**

**Figure S8. Density functional theory (DFT) analysis of defect states of Na-ZnO.**

**(a)** Charge-state transition levels relative to the conduction band (CB) and valence band (VB), revealing that the Na_Zn_-V_o_ coupled defect state lies approximately 1.3 eV below the conduction band, functioning as an effective carrier trap **(b)** Charge-density distributions of Na_Zn_-V_o_ coupling defect states.

**Table S1. Comparison of representative stress‑driven mechanoluminescence (ML) materials and this work.**

Comparison of host composition, unit‑cell complexity, luminescent centers, ML emission color, and representative emission wavelengths for reported stress‑driven ML materials, highlighting the earth‑abundant, rare‑earth‑free, and red‑to‑near‑infrared emitting characteristics of ZnO in this work.

| **Host composition** | **Number of atoms in the unit cell** | **Luminescent center** | **ML color** | **Ref** |
| --- | --- | --- | --- | --- |
| **ZnO** | **4** | **None** | **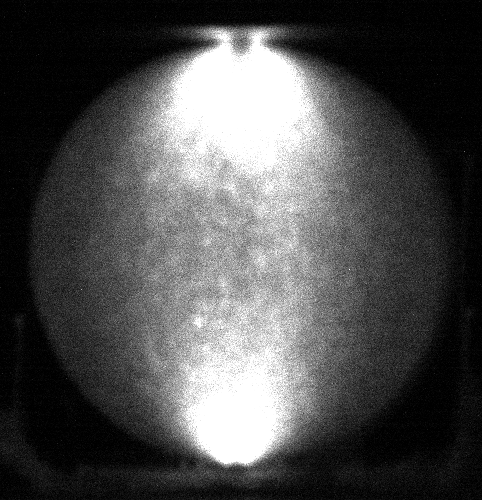Red-NIR**  650-900 nm | **This**  **work** |
| SrMg_2_(PO_4_)_2_ | 52 | Eu^2+^, RE | **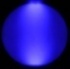**Blue  380-500 nm | ^[1]^ |
| SrCaAl_2_Si_2_O_8_ | 104 | Eu^2+^, RE | **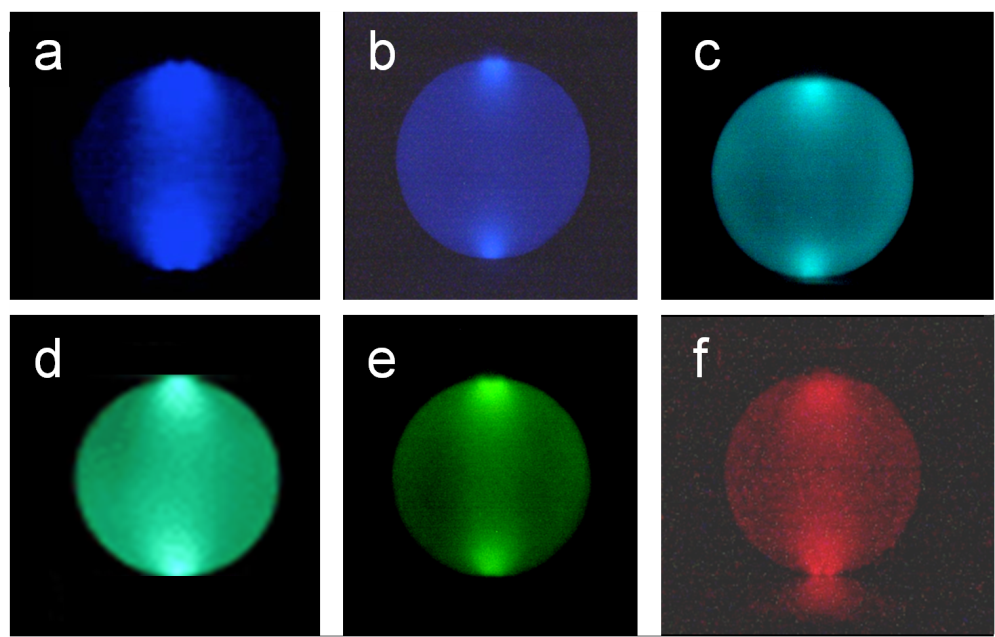**Blue  380-600 nm | ^[2]^ |
| CaYAl_3_O_7_ | 24 | Eu^2+^, RE | **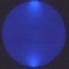**Blue  380-500 nm | ^[3]^ |
| SrCaMgSi_2_O_7_ | 24 | Eu^2+^, RE | **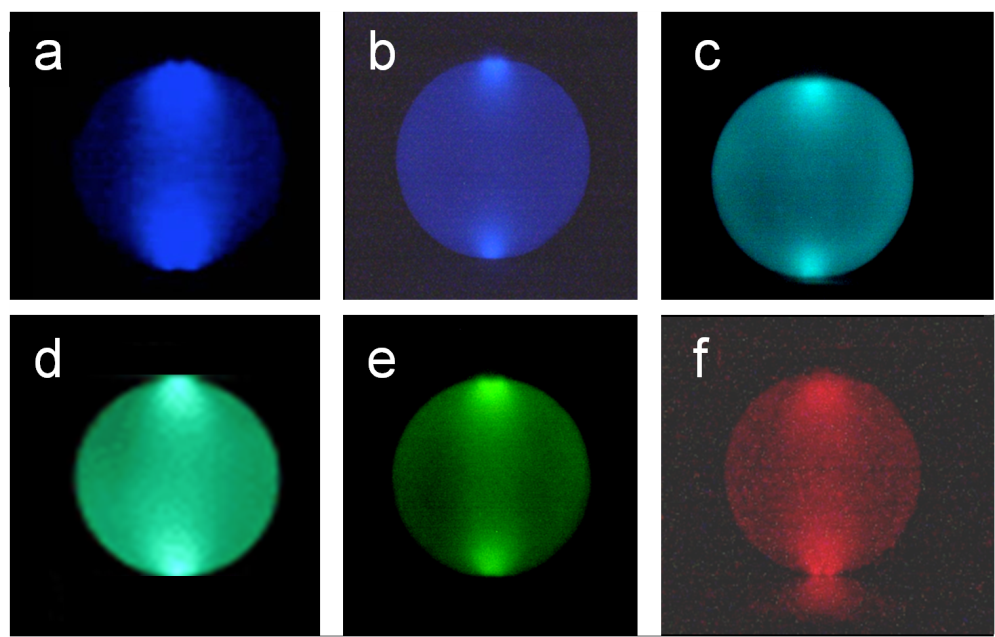**Light  Blue  420-550 nm | ^[4]^ |
| Ca_2_MgSi_2_O_7_ | 24 | Eu^2+^, RE | **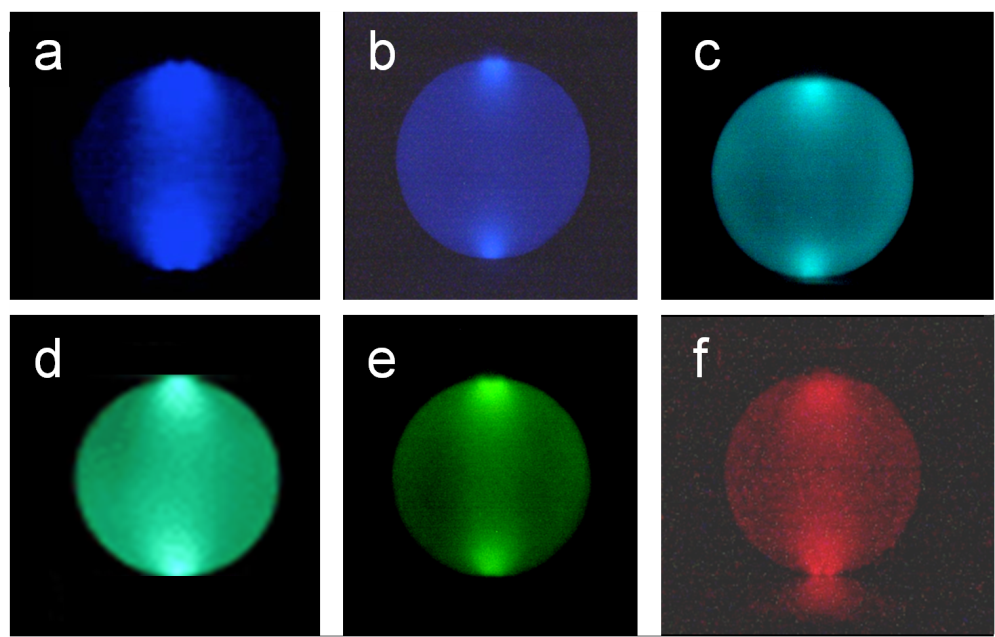**Green  480-600 nm | ^[4]^ |
| SrAl_2_O_4_ | 28 | Eu^2+^, RE | **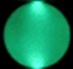**Green  450-600 nm | ^[5]^ |
| Sr_2_SiO_4_ | 28 | Eu^2+^, RE | **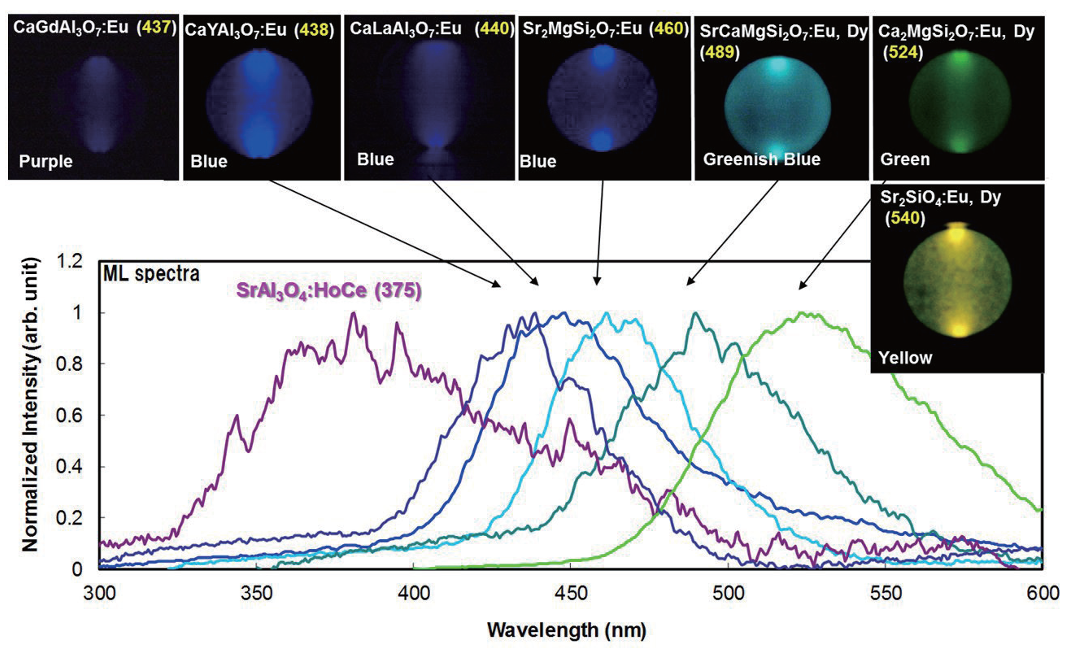**Yellow  400-650 nm | ^[6]^ |
| Sr_3_Sn_2_O_7_ | 48 | Sm^3+^, RE | **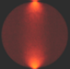**Red  560-700 nm | ^[7]^ |
| (Li,Na)NbO_3_ | 10 | Pr^3+^, RE | **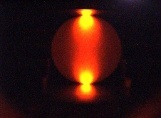**Red  600-650 nm | ^[8]^ |
| (Ba,Ca)TiO_3_ | 5 | Pr^3+^, RE | **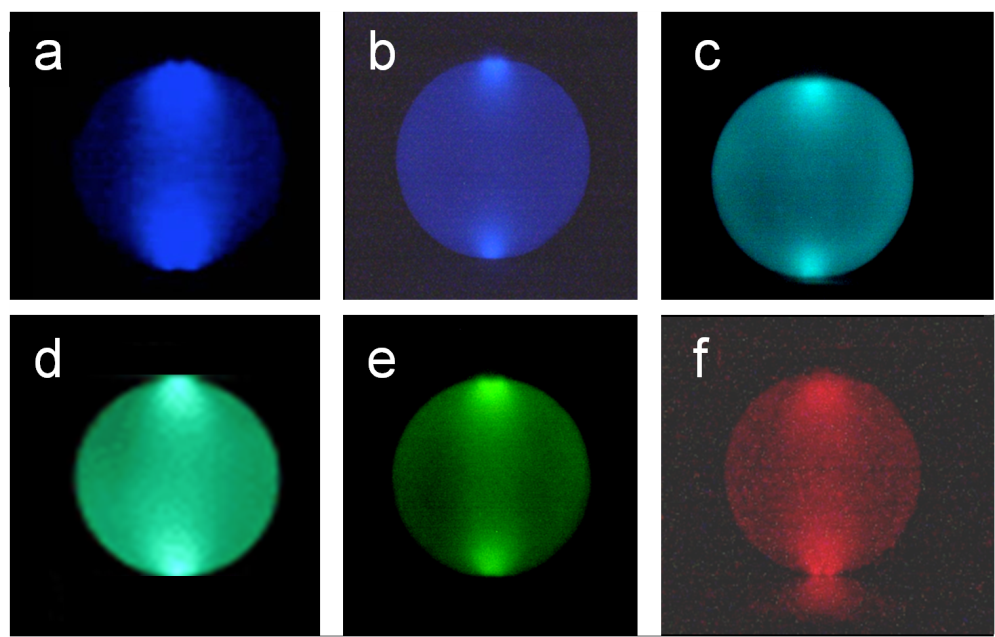**Red  600-650 nm | ^[9]^ |
| Sr_3_Sn_2_O_7_ | 48 | Nd^3+^, RE | **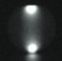**NIR  800-1500 nm | ^[10]^ |
| SrAl_2_O_4_ | 28 | Cr^3+^, Eu^2+^, Nd^3+^ | NIR  800-1550 nm | ^[11]^ |
| CaZnOS | 8 | Mn^2+^ | Yellow/Red  550-700 nm | ^[12]^ |
| Sonosensitized hydrogen-bonded organic framework (HOF) nanoparticles | > 100 | Molecules | Blue/Red  400-700 nm | ^[13]^ |

**Video S1. Near‑infrared mechanoluminescence (NIR ML) through biological tissue.**

Demonstration of stress‑induced NIR ML emission from Na–ZnO observed through biological tissue under compressive loading.

**Video S2. Stress-induced NIR ML imaging through pork tissue.**

Pseudo-color video showing stress-induced NIR ML from Na-ZnO transmitted through pork tissue during mechanical loading.

**Video S3. Stress-driven ML measurement.**

Experimental setup and measurement procedure for evaluating ML responses under controlled mechanical stress.

**References**

[1] S. Kamimura, H. Yamada, C. N. Xu, *J. Lumin.* **2012**, *132*, 526. https://doi.org/10.1016/j.jlumin.2011.09.033

[2] L. Zhang, C. N. Xu, H. Yamada, N. Bu, *J. Electrochem. Soc.* **2010**, *157*, J50. https://doi.org/10.1149/1.3274879

[3] H. Zhang, H. Yamada, N. Terasaki, C. N. Xu, *J. Electrochem. Soc.* **2008**, *155*, J128. https://doi.org/10.1149/1.2890856

[4] H. Zhang, H. Yamada, N. Terasaki, C. N. Xu, *J. Electrochem. Soc.* **2008**, *155*, J55. https://doi.org/10.1149/1.2816215

[5] C. N. Xu, T. Watanabe, M. Akiyama, X. G. Zheng, *Appl. Phys. Lett.* **1999**, *74*, 2414. https://doi.org/10.1063/1.123865

[6] Y. Mori, H. Zhang, H. Yamada, C. N. Xu, *Annual Meeting of The Ceramic Society of Japan* **2012**, 1P091. https://doi.org/10.14853/pcersj.2012s.0.198.0

[7] S. Kamimura, H. Yamada, C. N. Xu, *Appl. Phys. Lett.* **2012**, *101*, 091113. https://doi.org/10.1063/1.4749807

[8] H. Hara, C. N. Xu, R. Wang, X. G. Zheng, M. Nishibori, E. Nishibori, *J. Ceram. Soc. Jpn.* **2020**, *128*, 518. https://doi.org/10.2109/jcersj2.20015

[9] X. Wang, C. N. Xu, H. Yamada, K. Nishikubo, X. G. Zheng, *Adv. Mater.* **2005**, *17*, 1254. https://doi.org/10.1002/adma.200401406

[10] D. Tu, C. N. Xu, S. Kamimura, Y. Horibe, H. Oshiro, L. Zhang, Y. Ishii, K. Hyodo, G. Marriott, N. Ueno, N. Ueno, X. G. Zheng, *Adv. Mater.* **2020**, *32*, 1908083. https://doi.org/10.1002/adma.201908083

[11] Y. Fujio, C. N. Xu, N. Terasaki, *J. Electrochem. Soc.* **2021**, *168*, 047508. https://doi.org/10.1149/1945-7111/abf5f8

[12] C. Li, C. N. Xu, L. Zhang, H. Yamada, Y. Imai, *J. Vis.* **2008**, *11*, 329. https://doi.org/10.1007/BF03182201

[13] W. Wang, Y. Shi, W. Chai, K. W. K. Tang, I. Pyatnitskiy, Y. Xie, X. Liu, W. He, J. Jeong, J.-C. Hsieh, A. R. Lozano, B. Artman, X. Shi, N. Hoefer, B. Shrestha, N. B. Stern, W. Zhou, D. W. McComb, T. Porter, G. Henkelman, B. Chen, H. Wang, *Nature* **2025**, *638*, 401. https://doi.org/10.1038/s41586-024-08401-0
